# Comparative Analysis of the Ovary Transcriptome among Wanyue Black and Yorkshire Gilts Using RNA-Seq

**DOI:** 10.3390/vetsci11030115

**Published:** 2024-03-04

**Authors:** Huibin Zhang, Shuo Chen, Yangguang Liu, Fan Xie, Haoyu Wen, Shiming Zhao, Xianrui Zheng, Yueyun Ding, Zongjun Yin, Xiaodong Zhang

**Affiliations:** 1 College of Animal Science and Technology, Anhui Agricultural University, Hefei 230036, China; zhanghuibin1997@126.com (H.Z.); 18726309839@163.com (S.C.); lyg236200@163.com (Y.L.); fanxie1999@163.com (F.X.); a2824792003@163.com (H.W.); zhaoshiming175@126.com (S.Z.); zxr07sk1@163.com (X.Z.); dingyueyun@ahau.edu.cn (Y.D.); 2 Key Laboratory of Local Animal Genetic Resources Conservation and Bio-Breeding of Anhui Province, Hefei 230036, China

**Keywords:** gilt, puberty, reproductive hormones, ovary, RNA-seq

## Abstract

**Simple Summary:**

The gene expression of ovarian transcriptome varies among different gilt breeds. Here, we conducted a comparative analysis of ovarian and serum hormone levels during puberty onset between the indigenous Chinese Wanyue Black pig breed and the imported Yorkshire breed. Our findings revealed a significant enrichment of differentially expressed genes involved in the reproduction, ovarian follicle development, and hormone secretion signaling pathways. Additionally, employing bioinformatics analysis, we identified multiple candidate genes potentially involved in the regulation of the ovaries. Our results provide new insights on gilt ovary gene expressions among Chinese indigenous pig breeds versus Yorkshire which can serve as useful genetic tools to develop the gene assays for trait-associated studies.

**Abstract:**

Pubertal genetic variations between the indigenous Chinese Wanyue Black pig breed and the imported Yorkshire breed significantly impact their reproductive capacity. In order to identify the differentially expressed genes, gene networks, and metabolic pathways in ovary transcriptome of gilts, the serum hormone levels were analyzed by ELISA, and RNA-seq was performed to analyze ovarian genes. Our results reveal higher estradiol (E2) levels in Wanyue black gilts compared to Yorkshire gilts, while Yorkshire gilts exhibit elevated progesterone (P4) and GnRH levels. We identified a total of 154 differentially expressed genes (DEGs), with 87 up-regulated and 67 down-regulated genes in the Wanyue black gilts ovaries compared to the Yorkshire gilts. GO enrichment analysis unveiled the participation of DEGs in processes such as “Reproduction”, “Reproductive system development”, and “Ovarian follicle development”. Moreover, KEGG enrichment analysis revealed the involvement of DEGs in multiple signaling pathways associated with hormone biosynthesis and puberty, encompassing “Steroid hormone biosynthesis”, “Estrogen signaling pathway”, and “Prolactin signaling pathway”. The subsequent bioinformatics analysis identified nine functional genes that potentially contribute to the disparity in ovaries between Wanyue black gilts and Yorkshire gilts. This study offers significant insights into the endocrine and genetic aspects of pubertal development in gilts.

## 1. Introduction

The productivity and economic sustainability of swine production rely significantly on the reproductive performance of the sow, underscoring the importance of preserving and enhancing this facet. Puberty onset can be identified by the initial estrus exhibited by the sow, indicating her attainment of fertility [1]. Puberty timing is primarily influenced by factors such as breed, weight, nutrition, and exposure to boars [2]. The age of puberty, to a certain extent, impacts the lifelong reproductive performance of sows. Li et al. reported that an early onset of puberty resulted in early mating and farrowing in culled females, and rising age at puberty increases the age at first mating/farrowing and reduces parity at culling in females [2]. Consequently, a suitable modification of the age of puberty onset can offer novel possibilities for enhancing the litter rate.

The hypothalamic–pituitary–ovarian (HPO) axis plays a pivotal role in initiating puberty, as evidenced by prior investigations [3,4], and modulating this axis holds promise as an effective therapeutic approach for addressing abnormal puberty [5]. The ovaries, as integral components of the female reproductive and endocrine systems, play a direct role in facilitating follicle maturation and synthesizing the steroids and peptide hormones crucial for initiating puberty, which profoundly influences reproductive performance [2,6,7,8]. With the onset of puberty, substantial changes occur in the endocrine function of animals. For instance, hormone levels secreted by hypothalamus, the pituitary gland, and ovaries change [9,10,11,12]. The hypothalamus secretes gonadotropin-releasing hormone (GnRH). GnRH stimulates the pituitary gland to secrete follicle-stimulating hormone (FSH) and luteinizing hormone (LH) [9]. These hormones regulate puberty in mammals. They influence gonadal folliculogenesis, ovulation, and sex steroid production [10]. In the last 14 days of the prepubertal stage, researchers observed an increase in LH and estradiol (E2) levels in Meishan gilts. In contrast, the levels of FSH did not change. However, in the pubertal stage, the levels of LH, FSH, and progesterone (P4) were higher [11]. In a recent study, hormonal indexes were identified as predictors of porcine reproductive traits and puberty [12]. This reveals the importance of reproductive hormones for puberty and reproductive traits in pigs.

The mechanism governing the initiation of puberty is remarkably intricate and encompasses the intricate regulation of hormone secretion, follicular growth and development, and ovarian function by a multitude of genes. Presently, a multitude of scholars have utilized candidate genes and molecular markers to explore the genetic mechanisms underlying mammalian puberty. Seminara and Messager [13] demonstrated that the GPR54 gene serves as a pivotal regulator of puberty. Additionally, polymorphic variants in genes such as KISS-1 [14], ER-α [15], and SHBG [16], among others, have been associated with mammalian puberty. Research findings have unveiled four gene loci (CTTNBP2NL, FRS2, KANK4, and KATNAL1) displaying robust indications of selection and functional correlation with puberty [17]. In the ovaries of porcine species during the proestrus and estrus stages, a comprehensive set of 2167 genes manifested differential expression, encompassing ITGA3, ITGA5, ITGA11, and ITGB3, which hold potential associations with follicular growth and oocyte maturation [18]. Chu et al. [19] conducted RNA sequencing (RNA-seq) to ascertain novel differentially expressed genes (DEGs) that potentially participate in the regulation of estrus, and these genes were discovered to be engaged in multiple signaling pathways, including ovarian steroidogenesis and steroid biosynthesis. In summary, the utilization of RNA-seq presents novel insights into the identification of potential candidate genes and molecular markers that play a pivotal role in modulating the timing of puberty.

Chinese indigenous pig breeds exhibit shared biological characteristics, including increased litter size and distinct estrus behaviors [20]. In comparison to Landrace and Large White breeds, sows of these Chinese breeds attain puberty at an earlier stage, manifest extended behavioral estrus periods, and display marginally shorter estrus cycles [21,22]. The Wanyue Black pig (WYB) represents a novel, superior-quality, lean breed that emerged from a collaborative endeavor between Anhui Agricultural University and Resource Seed Conservation Enterprises. The lineage of the WYB pig consists of 37.5% Huai pig, a native breed of China, and 37.5% Beijing Black pig, another domestically bred variety, with the remaining 25% derived from the imported breed, Duroc. Female WYB gilts attain maturity at approximately six months, presenting a contrast to the customary seven-month period observed in other East Asian domestic pigs. It was reported that age at puberty in Duroc, Landrace, and Yorkshire gilts was varied in a certain southern China farm. The puberty of Duroc gilts averaged 228.93 days, Landrace gilts took 221.45 days, and Yorkshire gilts took 229.31 days [2]. As mentioned earlier, reproductive endocrine and genetic factors assume a pivotal role in the regulation of puberty. However, a dearth of studies currently exists that investigate the gilt ovary gene expression difference between Chinese indigenous pig breeds and the Yorkshire breed. In order to examine the differences in reproductive hormones and ovarian genes among different pig breeds, we performed an analysis of serum hormone levels and ovarian gene expression profiles in pubertal sows of WYB gilts and Yorkshire (YS) gilts. We identified genes that potentially impact the regulation of gilt ovary, along with biological processes and pathways that could facilitate the identification of molecular genetic markers linked to swine ovarian development.

## 2. Materials and Methods

### 2.1. Animals and Sample Collection

**Figure 1:**
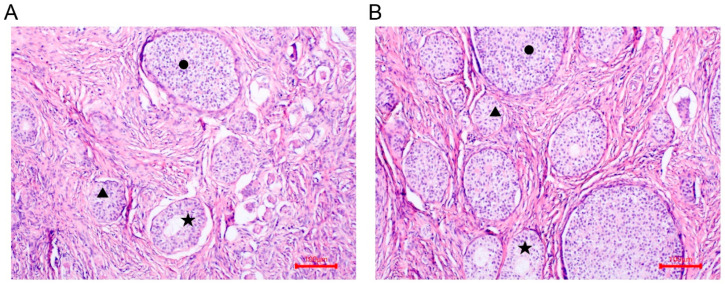
Representative images of WYB (**A**) and YS (**B**) ovaries. ▲ Primary follicle; ● Corpus luteum; ★ Secondary follicle.

Six gilts, consisting of three Wanyue Black pigs (aged 6.5 month old) and three Yorkshires (aged 7 month old), were provided with identical conditions on pig farms located in Anhui Province. Individual natural estrus was determined by observing the standing reflex and conducting the back-pressure test, along with the observation of vulvar redness [23], and meanwhile several mature follicles and corpus luteum were observed in ovaries (Figure 1) [24]. Prior to euthanasia, six blood samples were collected from the jugular vein of pubertal gilts. The collected blood (20 mL) was initially stored at 4 °C, followed by centrifugation at 3000 rpm for 10 min to isolate the serum, which was subsequently stored at −20 °C. Immediately after blood collection, the ovaries of the gilts were extracted, and any excess adipose tissue was excised. The ovaries were promptly frozen in liquid nitrogen and stored at −80 °C until they were required for subsequent analysis. In order to ensure result accuracy, the ovaries selected for sequencing were exclusively obtained from the right side of each gilt.

### 2.2. Serum Hormone Detection

The preserved serum samples were retrieved, and FSH (follicle stimulating hormone), LH (luteinizing hormone), E2 (estrogen), P4 (progesterone), GnRH (gonadotropin-releasing hormone), and PRL (prolactin) levels were measured by Elisa kit (Keshun Biotechnology Co., Ltd., Shanghai, China) in accordance with the manufacturer’s instructions. For the porcine FSH and LH Elisa kits, the assay sensitivity was <0.1 mIU/mL, and the coefficient of variation within and between plates was <15%. For the Porcine E2, GnRH, PRL Elisa kits, the assay sensitivity was <0.1 ng/mL and the coefficient of variation was <15%. For the Porcine P4 Elisa kit, the assay sensitivity was <0.1 pmol/mL and the coefficient of variation was <15%. Briefly, the Standards were diluted serially and test samples were incubated in an ELISA plate. The absorbance value (OD) at a wavelength of 450 nm was measured using a spectrophotometer. Subsequently, the concentration of reproductive hormones was determined using a standard curve. Each assay was performed in triplicate.

### 2.3. RNA Extraction, Library Construction, and Sequencing

Total RNA was extracted and purified from the entire ovarian tissues of gilts using TRIzol reagent (Invitrogen, Carlsbad, CA, USA) following the manufacturer’s instructions. Subsequent to extraction, the RNA samples were subjected to 1% agarose gel electrophoresis to evaluate potential contamination and degradation. The NanoPhotometer^®^ spectrometer (IMPLEN, Westlake Village, CA, USA) was used to assess the purity and concentration of the RNA samples. Moreover, the integrity and quantity of the RNA were meticulously evaluated using the RNA Nano 6000 assay kit on the Bioanalyzer 2100 system (Agilent Technologies, Santa Clara, CA, USA). RNA samples with an OD260/280 absorbance ratio ranging from 1.8 to 2.0 and an RNA integrity number (RIN) of 7.0 or higher were chosen for further experimental analyses.

The cDNA library was prepared for sequencing using the cDNA samples obtained from Wanyue Black and Yorkshire gilts. Briefly, total RNA samples were treated with DNase I to remove any genomic DNA contamination. Next, mRNA was isolated using oligo (dT)-attached magnetic beads and fragmented into smaller fragments using an appropriate temperature fragmentation buffer. Random primers were used for cDNA synthesis, followed by terminal repair and ligation. The resulting cDNA underwent PCR amplification, and the non-circularized linear DNA molecules were digested, resulting in the final library. Subsequently, the library was sequenced on the NovaSeq6000 platform (Novogene Bioinformatics Technology Co., Ltd., Beijing, China).

### 2.4. Analysis of RNA-Seq Data

The raw reads were subjected to initial processing using custom Perl scripts to filter out low-quality reads, reads containing adaptors, and reads harboring poly N sequences. This meticulous step involved removing reads that contained adaptors, had low quality, or harbored poly N sequences. The clean data was assessed for Q20, Q30, and GC contents, and the remaining high-quality clean reads were aligned to the swine reference genome *sus scrofa*(pig)-11.1 (https://ftp.ensembl.org/pub/release-109/gtf/sus_scrofa/, accessed on 12 December 2022) using HISAT2 software (v2.2.1, http://github.com/infphilo/hisat2/, accessed on 12 December 2022). Transcript assembly, quantification of gene expression, and estimation of gene expression abundance were performed using the StringTie software (v2.2.1, http://ccb.jhu.edu/software/stringtie, accessed on 12 December 2022). Additionally, Gffcompare was used to scan the genomic landscape, detect gene annotations, and analyze transcript assemblies. The DESeq2 R package was utilized to identify differentially expressed genes (DEGs) between the two investigated groups. Gene expression quantification was meticulously performed using the Fragments Per Kilobase of transcript per Million mapped reads (FPKM) method, enabling a comprehensive assessment of gene activity. Genes with |log2Fold Change| ≥ 1 and *p*-adjust ≤ 0.05 were labeled as differentially expressed.

### 2.5. Bioinformatics Analysis

Gene Ontology (GO) enrichment and Kyoto Encyclopedia of Genes and Genomes (KEGG) pathway enrichment analysis of the differentially expressed genes (DEGs) were performed using the clusterProfiler R package. The DEGs were subsequently categorized into Biological Process, Molecular Function, and Cellular Component categories. GO terms with a *P*-adjust ≤ 0.05 and KEGG pathways with a *p*-value ≤ 0.05 are generally considered statistically significant. A protein–protein interaction (PPI) network of the differentially expressed genes was constructed by submitting gene symbols to the STRING database (v2.2.1, http://string-db.org/, accessed on 16 December 2022) and visualized using Cytoscape (v3.8.2, https://cytoscape.org/, accessed on 16 December 2022). A minimum composite score of 0.4 was utilized for the analysis.

### 2.6. Quantitative Real-Time RT-PCR (qRT-PCR)

To validate the accuracy of the sequencing results, a subset of differentially expressed genes (DEGs) was selected for verification using quantitative real-time polymerase chain reaction (qRT-PCR). Total RNA was reverse from transcribed to cDNA using the PrimeScript^TM^ RT reagent kit (Takara, Osaka, Japan). qRT-PCR was performed using SYBR Premix Ex Taq kit (TaKaRa) on a Bio-Rad CFX96 Real-Time Detection System (BioRad, Hercules, CA, USA). The 20 μL reaction solution contained 9 μL SYBR, 2 μL cDNA, 1 μL each of the forward and reverse primers, and 7 μL ddH_2_O. 

The qRT-PCR amplification protocol comprised a hot start at 95 °C for 5 s and 60 °C for 30 s followed by 40 cycles, 65 °C for 5 s, and 95 °C for 5 s. The 2^−ΔΔCt^ method was used to calculate the relative expression levels of genes, using β-actin as an internal control. All primer sequences are listed in Table 1. 

### 2.7. Statistical Analysis

The data analysis was performed using GraphPad Prism software (version 5.0, La Jolla, CA, USA). The significance of the difference between the two groups was assessed using Student’s *t*-test. The data were presented as the mean  ±  SEM, and a significance level of *p*  <  0.05 was considered statistically significant.

### 2.8. Statement of Use of AI or AI-Assisted Technologies

The English polish and logic of the article were completed with the help of GPT-4, a language model of OpenAI, and the text is free of any plagiarized, falsified, or fabricated material.

## 3. Results

### 3.1. Levels of Reproductive Hormones

**Figure 2:**
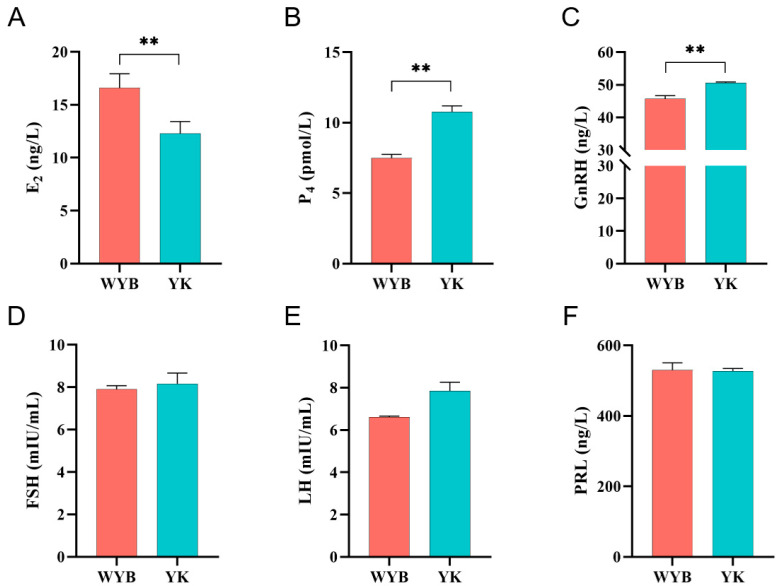
Serum reproductive hormone levels in WYB and YK gilts. (**A**) Serum E2, (**B**) P4, (**C**) GnRH, (**D**) FSH, (**E**) LH, and (**F**) PRL levels in Wanyue Black gilts and Yorkshire gilts. Values represent mean  ±  standard error. N = 3, ** *p* < 0.01.

As shown in Figure 2, WYB gilts exhibited significantly higher levels of serum estradiol (E2) compared to YK gilts (*p* < 0.01). Conversely, the levels of serum progesterone (P4) and gonadotropin-releasing hormone (GnRH) were significantly lower (*p* < 0.01). However, there was no significant difference (*p* > 0.05) in the levels of follicle-stimulating hormone (FSH), luteinizing hormone (LH), and prolactin (PRL) in the serum between YK and WYB gilts.

### 3.2. The Summary of RNA Sequencing Data

Six cDNA libraries (WYB1, WYB2, WYB3, YK1, YK2, YK3) were generated from ovarian tissues of Wanyue Black and Yorkshire gilts, and subsequently subjected to sequencing using the Illumina NovaSeq 6000 platform. WYB and YK refer to Wanyue Black and Yorkshire gilts, respectively. On average, YK gilt ovaries produced 43.46 million raw reads, while WYB gilt ovaries yielded 45.06 million raw reads. The data reveals an exceptionally low overall sequencing error rate of 0.03%. GC content refers to the proportion of cytosine and guanine in the complete genome of an organism. Interestingly, the samples display an average GC content of 52%, suggesting that the sequencing result is credible. Furthermore, each individual sample demonstrates Q20 and Q30 values of approximately 95% and 90%, respectively. Therefore, these findings validate the reliability of the sequencing data. Following the removal of low-quality reads, an average of 42.33 million and 43.88 million clean reads were obtained and utilized for subsequent analysis (Table S1). The clean reads were subsequently aligned to the porcine reference genome using HISAT2 software (v2.2.1, http://github.com/infphilo/hisat2, accessed on 12 December 2022). The mapping rate of clean reads to the reference genome ranged from 92.5% to 93.92%, with 90.43% to 91.75% of the clean reads uniquely aligned to the porcine reference genome, while 1.93% to 2.27% of the sequences had multiple alignments to the reference genome. These results indicate that the RNA sequencing data were suitable for subsequent bioinformatics analysis (Table S1).

### 3.3. Identification and Analysis of DEGs

**Figure 3:**
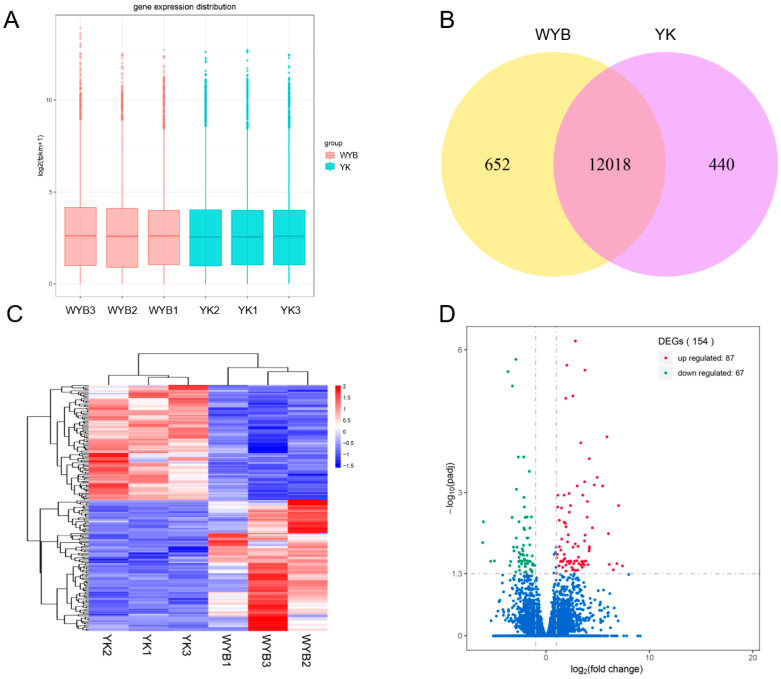
Expression profiles of mRNAs in WYB and YK gilts ovaries. (**A**) Boxplots showing the expression features of mRNAs. (**B**) Overlaps of expressed mRNAs. (**C**) Heatmap of mRNAs. (**D**) Volcano plot indicating up- and down-regulated mRNAs.

Gene expression levels were normalized using fragments per kilobase of transcript per million mapped reads (FPKM). The box plot of FPKM distribution revealed comparable gene expression patterns between YK and WYB gilts (Figure 3A). Based on the FPKM values, over 50% of the genes exhibited low expression levels (<1 FPKM), while approximately 25% of the genes displayed medium expression levels (1 FPKM to 15 FPKM), and around 13% of the genes exhibited medium to high expression levels (15 FPKM to 60 FPKM). Furthermore, a minority of genes exhibited higher expression levels (>60 FPKM). A total of 13,110 genes (FPKM > 1) were identified as expressed in the two gilt breeds. Among these, 12,018 genes were commonly expressed in both gilt breeds, while 652 genes were exclusively expressed in WYB gilts, and 440 genes were exclusively expressed in YK gilts (Figure 3B). Furthermore, the heatmap analysis revealed distinguishable gene expression patterns between YK and WYB, thus validating the reproducibility of gene expression within each group (Figure 3C). Subsequently, the DESeq2 software (v3.18, http://www.bioconductor.org/packages/release/bioc/html/DESeq2.html, accessed on 12 December 2022) was employed to identify differentially expressed genes. Then, a total of 154 DEGs were identified in the WYB vs. YK comparison (*P*.adj < 0.05, |log2Fold Change| ≥ 1.0), where 87 were up-regulated (|log2Fold Change| ranging from 1.02 to 7.40) and 67 were down-regulated (|log2 Fold Change| ranging from 1.06 to 6.12) in the WYB group compared to the YK group (Figure 3D).

### 3.4. Enrichment Analyses of DEGs

**Figure 4:**
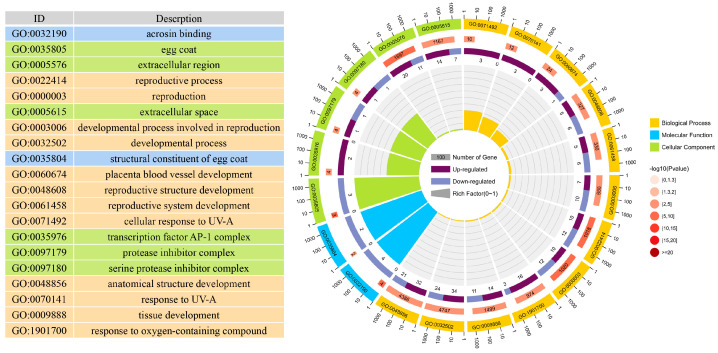
GO Analyses of DEGs in WYB and YK gilts ovaries, including top 20 enriched GO terms of the DEGs. Different colors represent different categories. The second circle represents the *p*-value and number of background genes in each category. The larger the number of genes, the longer the bar. The smaller the *p*-value, the darker the red color. The third circle represents the total number of foreground genes. The fourth circle represents the Rich Factor value of each category (the number of foreground genes in the category divided by the number of background genes). Each grid of the background auxiliary line represents 0.1.

To investigate the functional roles of the differentially expressed genes (DEGs), we performed Gene Ontology (GO) enrichment analysis on the 154 DEGs (Table S2). The GO annotations of these DEGs were classified into three categories, including 2615 terms for Biological Process (BP), 300 terms for Cellular Component (CC), and 480 terms for Molecular Function (MF). Figure 4 displays the top 20 GO terms, and a limited number of terms in each category were found to be statistically significant (P.adj < 0.05). Overall, only 10 terms were significantly enriched in the Cellular Component (CC) category, such as “egg coat”, “extracellular region”, “transcription factor AP-1 complex”, “protease inhibitor complex”, and “serine protease inhibitor complex”. Additionally, eight terms showed significant enrichment in the Molecular Function (MF) category, including “acrosin binding”, “structural constituent of egg coat”, “signaling receptor regulator activity”, “cytokine activity”, and “peptidase regulator activity”. However, a total of 25 Biological Process (BP) terms were found to be significantly enriched in the DEGs, several of which are known to be involved in reproduction, reproductive system development, and ovarian follicle development. These terms included “reproductive process”, “developmental process involved in reproduction”, “reproductive structure development”, and “gonad development” (Table 2). Our analysis revealed that ZP3, BMP15, CEBPB, and VGF were implicated in ovarian follicle development, reproduction, and reproductive system development. Additionally, FOSL1, WNT2, JUNB, KITLG, and SOCS3 were specifically associated with reproduction and reproductive system development.

**Figure 5:**
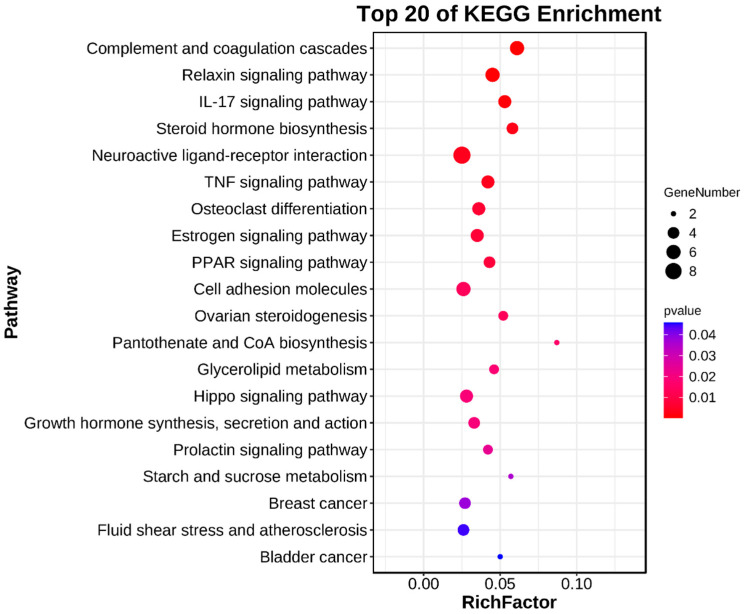
Significantly enriched KEGG pathways of DEGs. The abscissa represents the Rich factor, and the ordinate represents the pathway name.

Subsequently, KEGG enrichment analysis was performed on the differentially expressed genes (DEGs) using Figure 5 and Table S3 as references. In total, 21 pathways displayed significant enrichment (*p* < 0.05), encompassing various pathways such as “Complement and coagulation cascades”, “Relaxin signaling pathway”, “IL-17 signaling pathway”, “Neuroactive ligand-receptor interaction”, “TNF signaling pathway”, “PPAR signaling pathway”, “Pantothenate and CoA biosynthesis”, and “Hippo signaling pathway”. Furthermore, several pathways were found to be associated with puberty, including “Steroid hormone biosynthesis”, “Estrogen signaling pathway”, “Ovarian steroidogenesis”, “Growth hormone synthesis, secretion and action”, and “Prolactin signaling pathway” (Table 3). Our analysis revealed that five genes, namely HSD3B1, AKR1D1, CYP11A1, UGT1A6, and BMP15, were enriched in two signaling pathways associated with steroidogenesis. Notably, within the signaling pathways associated with estrogen, growth hormone, and prolactin, we identified two genes, FOS and SHC2, that play a shared role in the regulation of these three pathways. Additionally, SOCS3 was specifically implicated in the growth hormone and prolactin signaling pathways.

### 3.5. PPI Network Analysis

**Figure 6:**
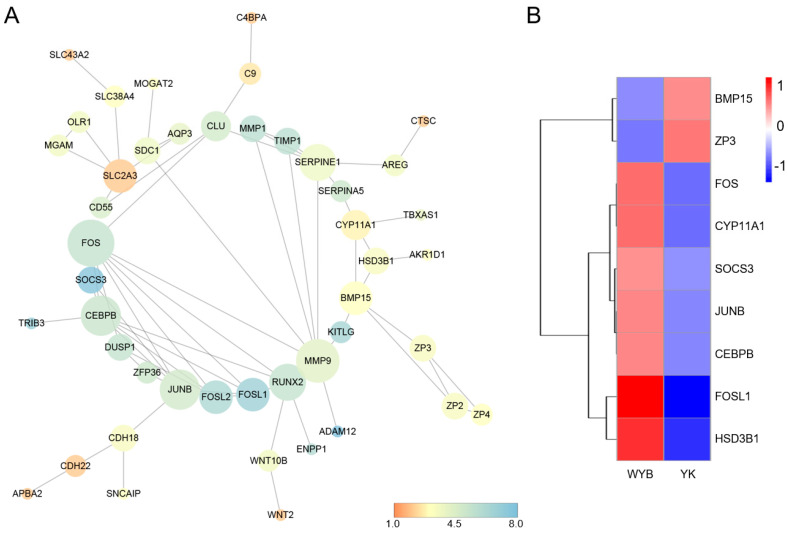
PPI network and hub gene screening. (**A**) PPI network for DEGs. (**B**) Heatmap of hub gene expression.

In order to identify pivotal genes involved in the regulation of puberty onset, we constructed a protein–protein interaction (PPI) network using the differentially expressed genes (DEGs) identified from the evaluation of WYB and YK ovaries (Figure 6A). To identify significant genes, we eliminated small networks with interaction scores below 0.4, along with their corresponding genes and nodes. Additionally, disconnected nodes were concealed within the network. Ultimately, a central PPI network was established, consisting of 46 nodes and 68 edges where the interaction score was equal to or greater than 0.4. The nodes within the network represent proteins, while the edges connecting two nodes indicate a potential functional association between the corresponding proteins. The color filling of the nodes represents neighborhood connectivity, and the node size reflects the degree and intensity of the interaction. The average node degree was calculated to be 2.96. As we conducted gene screening during the construction of the PPI network, genes that did not participate in the network construction were excluded. Consequently, we considered genes with a node degree higher than the average node degree (node degree > 2.96) in the present PPI network to be hub genes. Integrating the enrichment analyses of DEGs, we identified nine genes, namely FOS, CEBPB, JUNB, FOSL1, BMP15, CYP11A1, SOCS3, ZP3, and HSD3B1, which may contribute to the variations in puberty onset between WYB and YK gilts. To visualize the expression patterns of these genes, we generated a heatmap illustrating the differential expression of these genes in WYB and YK gilts (Figure 6B).

### 3.6. RNA-Seq Data Validation by RT-qPCR

**Figure 7:**
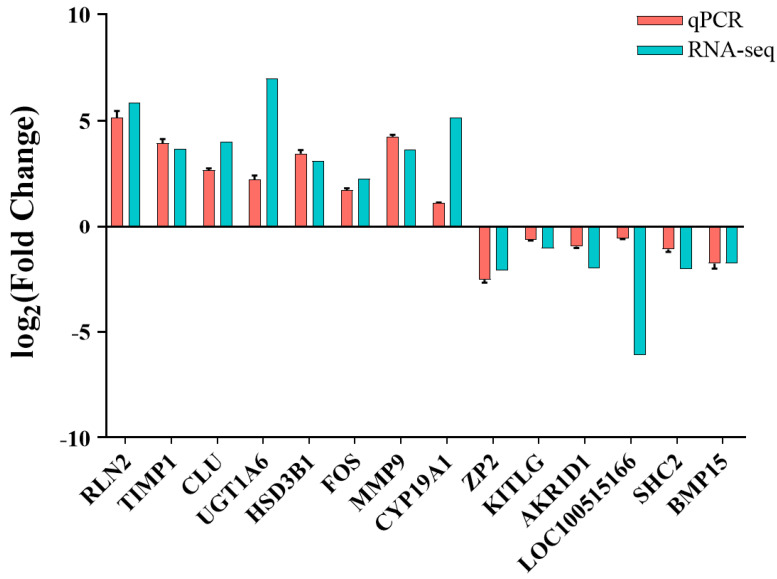
Validation of expression levels of DEGs by RT-qPCR, N = 3. The YK group was set as the reference group, and log_2_(Fold Change) is the relative expression of genes in the WYB group.

To validate the accuracy of the RNA-seq data, we randomly selected 14 differentially expressed genes (DEGs) and assessed their average expression levels in WYB and YK gilt ovaries using RT-qPCR, respectively. We confirmed the expression levels of these genes in all six ovaries of YK and WYB gilts. Figure 7 demonstrates that the expression patterns of the 14 DEGs were in agreement with the RNA-seq data, thereby confirming the reliability of the RNA-seq results.

## 4. Discussion

Enhancing the reproductive performance of pigs in livestock production directly contributes to the enhancement of breeding profitability. Precocious puberty is a notable reproductive characteristic observed in indigenous pig breeds in China. Therefore, investigating the regulatory mechanisms underlying precocious puberty in livestock and poultry is of paramount importance. The Chinese Wanyue Black pig exhibits distinctive precocious puberty traits, with gilts attaining sexual maturity at approximately 6 months of age. This breed serves as an ideal experimental model for investigating sexually precocious puberty traits. Ovarian development significantly influences the timing of sexual maturity in female animals, thereby playing a pivotal role in determining their reproductive performance. Puberty in mammals encompasses crucial processes, including hormone secretion, follicular development, and ovulation. To examine the endocrine disparities between the two pig breeds during puberty, we employed ELISA to measure the serum levels of FSH, E2, LH, P4, GnRH, and PRL in pubertal gilts. During puberty initiation, GnRH levels rise, activating the hypothalamic–pituitary–ovarian (HPO) axis. Previous studies have proposed the involvement of GnRH in puberty regulation, as it stimulates ovarian growth and initiates primiparity through its influence on the expression of other fertility hormones and their receptor genes [25]. Significantly higher GnRH levels were observed during puberty in Yorkshire pigs compared to Wanyue Black gilts (*p* < 0.01). This difference could be attributed to the potential occurrence of a missed pre-pubertal peak. Furthermore, GnRH requires time for metabolism, which could contribute to the increased levels observed in Yorkshire pigs during puberty. Thompson and Garza [26] reported an increase in LH, FSH, and E2 levels during pre-puberty. Consistent with these findings, our data revealed a significantly higher serum E2 level in Wanyue Black pigs compared to Yorkshire pigs (*p* < 0.01), while the P4 level was significantly lower in Wanyue Black pigs than in Yorkshire pigs (*p* < 0.01). Generally, individual reproductive hormones cannot independently govern the progression of puberty; they interact with one another to regulate its onset [24]. Although the reproductive hormones PRL, LH, and FSH did not exhibit significant differences between the two pig breeds (*p* > 0.05), further investigation is warranted to explore the specific regulatory mechanisms, considering the limited availability of studies on these hormones during puberty onset in animals.

Given the extensive influence of genes on serum hormone metabolism and ovarian function in animals, we conducted a comprehensive investigation of differentially expressed genes (DEGs) in the ovaries of Wanyue Black and Yorkshire gilts during the puberty stage. A total of 13,110 genes were identified through RNA-seq analysis of the ovaries from Wanyue Black and Yorkshire gilts, and subsequent screening identified 154 DEGs that met our predefined criteria. In order to gain deeper insights into the underlying biological processes associated with ovarian development, we performed functional classification of the DEGs identified in the ovarian tissues of pubertal gilts.

The GO enrichment results revealed a significant enrichment phenomenon between Wanyue Black and Yorkshire, particularly in terms of Biological Processes related to reproduction, including “reproductive process”, “reproduction”, “reproductive structure development”, “developmental process involved in reproduction”, “reproductive system development”, “gonad development”, and “ovarian follicle development”. Notably, HSD3B1, AKR1D1, CYP11A1, UGT1A6, and BMP15 exhibited higher prevalence in these processes. Furthermore, FOS, SHC2, and SOCS3 are implicated in the synthesis and secretion of specific hormones, including estrogen, growth hormone, and prolactin. It is postulated that the expression of these genes may be linked to the hormonal secretion levels in Wanyue Black gilts during puberty. KEGG analysis of DEGs unveiled several pathways associated with puberty, encompassing “Steroid hormone biosynthesis”, “Estrogen signaling pathway”, “Growth hormone synthesis, secretion, and action”, “Ovarian steroidogenesis”, and “Prolactin signaling pathway”. Following functional enrichment analyses of DEGs, we constructed a protein–protein interaction (PPI) network of DEGs. Nine pivotal genes, including FOS, CEBPB, JUNB, FOSL1, BMP15, CYP11A1, SOCS3, ZP3, and HSD3B1, were identified as significant regulators involved in reproductive hormone secretion, follicle development, and reproductive performance, potentially contributing to the disparities in ovarian development between Wanyue Black and Yorkshire gilts.

Differential expression analysis revealed lower expression levels of BMP15 and ZP3 mRNA in the ovaries of Wanyue Black gilts compared to Yorkshire gilts. Conversely, the remaining seven key genes, including FOS, CYP11A1, and HSD3B1, exhibited higher expression in Wanyue Black gilts. Previous studies have reported that FOS, a subunit of the AP-1 transcription factor, exerts a positive regulatory role in ovarian follicle development [27]. Additionally, inhibition of FOS affects the expression of genes involved in the periovulatory process, thereby influencing follicular development, ovulation, and luteinization [28]. Fos knockout mice exhibited arrested follicular development and reduced levels of FSH and LH [29]. Collectively, these studies suggest that the expression of FOS in the ovary is indispensable for ovulation and luteinization. Within our constructed PPI network, the FOS gene exhibited the highest node degree and is considered a crucial regulatory gene. Lu and Zhou [30] reported a significant elevation in mRNA levels of HSD3B1 and CYP19A1 in ground squirrel ovaries during the breeding season. Additionally, HSD3B1 is implicated in the hydroxylation metabolism of estrogen, a process closely associated with the reproductive capacity of animals [31]. CYP11A1 plays a pivotal role in ovarian development and function, as well as various other physiological processes in both sexes [32]. Previous studies have demonstrated that modulating the expression of CYP11A1 and HSD3B1 can stimulate luteal angiogenesis, mitigate fluctuations in sex hormones in mice, and confer benefits to pregnancy [33]. In accordance with the aforementioned findings, our study revealed a significantly higher mRNA expression of CYP11A1 and HSD3B1 in ovarian tissue of Wanyue Black gilts compared to Yorkshire gilts. The expression of ZP3 mRNA is lower in Wanyue Black gilts compared to Yorkshire gilts. Previous research has primarily focused on the role of the ZP gene family in regulating sperm binding to the oocyte during fertilization [34]. ZP mRNA expression has been associated with empty follicle syndrome and abnormal zona pellucida [35]. Additionally, it likely participates in regulating follicle size and plays a crucial role in female fertilization and reproductive control. The mRNA expression pattern of BMP15 in the two gilt breeds mirrors that of ZP3. BMP15 is a well-known paracrine factor secreted by oocytes [36] and primarily responsible for regulating the secretion of steroid hormones, cell mitosis, and follicle development [37]. Previous research has demonstrated a negative correlation between intrafollicular concentrations of BMP15 and follicle size [38]. Studies have revealed that the expression of BMP15 in human ovarian granulosa cells stimulates the production of estrogen [39]. However, our study found an inverse relationship between serum estrogen concentrations in Wanyue Black gilts and the expression level of BMP15 in ovaries. This discrepancy may be attributed to the involvement of BMP15 in the intricate BMP15/TGF-β/SMAD signaling pathway, which plays a role in the regulation of steroid hormone production [40]. This study identified differentially expressed genes, including FOS, CEBPB, JUNB, FOSL1, BMP15, CYP11A1, SOCS3, ZP3, and HSD3B1, in Wanyue Black and Yorkshire gilt ovaries. Nevertheless, additional research is necessary to elucidate the precise roles of these candidate genes in the regulation of ovarian development and puberty. We selected three Wanyue black and three Yorkshire gilts for the study, and the low number of animals and ovaries limited our research. Our study has certain limitations; in the subsequent research, we will increase the number of animals and expand the population of pigs to compare the differences in ovarian gene expression between the bred Wanyue Black pig, the native Chinese Black pig, and the introduced Yorkshire breed before and after the estrus initiation. This will allow us to identify potential candidate genes that may influence gilt ovarian development.

## 5. Conclusions

In this study, we first investigated the serum reproductive hormone levels during the puberty stage in Wanyue Black gilts and Yorkshire gilts. We found that E2 levels were higher in Wanyue Black gilts compared to Yorkshire gilts, while P4 and GnRH levels were higher in Yorkshire gilts. RNA-seq analysis revealed 154 DEGs in the ovaries. Enrichment analyses of DEGs showed that DEGs were involved in ovarian follicle development and steroid hormone biosynthesis, among other processes. Finally, we identified nine functional genes that may influence differences in the ovarian development between Wanyue Black gilts and Yorkshire gilts. Our results provide new insights on gilt ovary gene expression among indigenous Chinese pig breeds versus Yorkshire pigs.

## Figures and Tables

**Table 1:** Primer sequences used for RT-qPCR.

Gene Name	Forward Primer	Reverse Primer
β-actin	GGACTTCGAGCAGGAGATGG	AGGAAGGAGGGCTGGAAGAG
RLN2	CTGAGCCAACTTCCCAGAGA	CCACCCACAGACGGACTAAT
ZP2	CACTGGAGTGACTCGCTACT	GTCACGTGTGTGGCATTACA
TIMP1	GTTTCCCTGCACATCCATCC	TCTGGAAGCCCTTGTCAGAG
CLU	AAATCACTGCTCAGCTCCCT	ATCGTCTCATTGCACAAGCC
KITLG	CCTCGTGGAATGCATGGAAG	TGCCACCATCTCCAAATCCT
UGT1A6	CCCAACCCACTGTCCTATGT	CAGAGGAAGCCCTCTGACAA
AKR1D1	CCTCTCTTGCTTGGTTGCTC	GTTAGGCTGAGGGACTTGGT
HSD3B1	TTCAATCGCCACTTCGTGAC	CCAGGTCAGTGAGTCTTGGT
LOC100515166	AGATCGAGAGCCTGAACGAG	GCCTCATACTGCTCCCTCAT
SHC2	CATCCTGGGCAAGAGCAATC	GGCCATCAATGGAGATGCTG
FOS	AGCTGACTGACACACTCCAA	ATCAAGGGAAGCCACAGACA
MMP9	TTCTTCTCTGGACGCCAAGT	TTCACGTCGAACCTCCAGAA
BMP15	TTCACTTGGACTCTGGGCAT	ATTTGCAACACAGCCCAGTT
CYP19A1	TATCCTTGCACCCGGATGAG	AGCTAGCAAAGATGGGTGGT

**Table 2:** GO terms associated with reproduction and ovarian development.

GO Term	*P*.adj	Count	Genes
Reproduction	0.0081	22	SERPINA5; TDRD9; SALL1; MMP9; ZP3; FKBP6; ZP2; AREG; RXFP2; ZP4; CLDN11; TIMP1; BMP15; ACSL4; FOSL1; JUNB; DND1; WNT2; CEBPB; KITLG; SOCS3; VGF
Reproductive system development	0.0139	11	SALL1; ZP3; RXFP2; BMP15; FOSL1; JUNB; WNT2; CEBPB; KITLG; SOCS3; VGF
Ovarian follicle development	0.0397	4	ZP3; BMP15; CEBPB; VGF

**Table 3:** KEGG pathways associated with reproductive hormone.

KEGG Pathway	*p*	Count	Genes
Steroid hormone biosynthesis	0.0028	4	HSD3B1, AKR1D1, CYP11A1, UGT1A6
Estrogen signaling pathway	0.0071	5	FOS, KRT14, MMP9, SHC2, LOC100515166
Ovarian steroidogenesis	0.0133	3	HSD3B1, CYP11A1, BMP15
Growth hormone synthesis, Secretion, and action	0.0194	4	FOS, JUNB, SOCS3, SHC2
Prolactin signaling pathway	0.0236	3	FOS, SOCS3, SHC2

## Data Availability

All raw data during the current study are available in the NCBI BioProject (https://submit.ncbi.nlm.nih.gov/subs/bioproject) with accession number PRJNA1031772 (accessed on 25 October 2023).

## Supplementary Materials

The following supporting information can be downloaded at: https://www.mdpi.com/article/10.3390/vetsci11030115/s1, Table S1: Summary of sequencing reads aligned with the Sus Scrofa genome; Table S2: GO analyses of DEGs; Table S3: KEGG pathway analyses of DEGs.
